# Impact of a History of COVID-19 Infection on Postoperative Complications in Spinal Surgery: A Nationwide Cohort Study

**DOI:** 10.3390/jcm15020420

**Published:** 2026-01-06

**Authors:** Namhoo Kim, Joonoh Seo, Minae Park, Yoonjong Bae, Min Ho Lee, Byung Ho Lee, Si-Young Park, Kyung-Soo Suk, Seong-Hwan Moon, Hak-Sun Kim, Ji-Won Kwon

**Affiliations:** 1 Department of Orthopedic Surgery, Yonsei University College of Medicine, Seoul 03722, Republic of Korea; namhoo.kim@yuhs.ac (N.K.); mhlee164@yuhs.ac (M.H.L.); bhlee96@yuhs.ac (B.H.L.); drspine90@yuhs.ac (S.-Y.P.); sks111@yuhs.ac (K.-S.S.); shmoon@yuhs.ac (S.-H.M.); haksunkim@yuhs.ac (H.-S.K.); 2 Department of Orthopedic Surgery, Ewha Womans University College of Medicine, Seoul 07804, Republic of Korea; 01651s@eumc.ac.kr; 3 Department of Data Science, Hanmi Pharm. Co., Ltd., Seoul 05545, Republic of Korea; minae.park@hanmi.co.kr (M.P.); yoonjong.bae@hanmi.co.kr (Y.B.)

**Keywords:** COVID-19, operation, spinal surgery, postoperative complications, pneumonia

## Abstract

**Background/Objectives**: The postoperative implications of a history of coronavirus disease 2019 (COVID-19) in patients undergoing spinal surgery remain inadequately defined. This study investigated whether a history of COVID-19 is associated with increased postoperative complication risk and assessed how surgical timing after infection influences outcomes. **Methods**: Patients who underwent spinal surgery in 2020 were identified. Individuals with a confirmed history of COVID-19 were matched 1:3 by age and sex to uninfected controls. Patients were categorized by the interval between COVID-19 diagnosis and the index surgical date (≤1 month, >1–≤3 months, or >3–≤6 months). Postoperative pulmonary, cardiovascular, thromboembolic, infectious, and mortality outcomes were evaluated. Cumulative risks were estimated using Kaplan–Meier analysis, and adjusted hazard ratios (HRs) were determined using multivariable Cox proportional hazards models controlling for demographic and clinical factors. **Results**: Surgery performed ≤1 month after COVID-19 diagnosis was associated with significantly higher risks of pneumonia within 3 months (HR 3.91; *p* = 0.031) and 6 months postoperatively (HR 3.12; *p* = 0.049). Patients undergoing surgery >1–≤3 months after COVID-19 demonstrated increased risk of spinal and implant-related infections within 3 months (HR 2.12; *p* = 0.040), and this elevated risk persisted when surgery occurred >3–≤6 months after infection (HR 2.00; *p* = 0.022). No significant differences were observed in cardiovascular, thromboembolic, or mortality outcomes. **Conclusions**: A history of COVID-19 infection was associated with postoperative pneumonia and spinal and implant-related infections following spinal surgery. These findings suggest that prior COVID-19 infection may be a relevant consideration in perioperative risk assessment and surgical planning.

## 1. Introduction

Coronavirus disease 2019 (COVID-19), caused by Severe Acute Respiratory Syndrome Coronavirus 2 (SARS-CoV-2), has challenged global health systems in an unprecedented manner. Since late 2019, more than 700 million confirmed cases and approximately 7 million deaths have been recorded worldwide [1]. Despite widespread vaccination efforts and the development of novel therapeutics, SARS-CoV-2 continues to resurge in waves, and a growing proportion of the global population has been exposed, making truly “COVID-naïve” individuals increasingly rare. Beyond the direct morbidity and mortality associated with infection, the pandemic profoundly disrupted routine healthcare delivery. Hospitals worldwide faced resource shortages, staff redeployment, and overcrowding, leading to widespread postponement or cancellation of elective procedures, including spinal surgery, which often requires substantial resources, anesthesia, postoperative care, and, in some cases, prolonged hospitalization. These disruptions not only delayed essential care but may also have altered the characteristics of patients undergoing surgery, perioperative management practices, and ultimately surgical outcomes.

In spinal surgery, a field in which timing, comorbidities, and perioperative risk interact, these changes raise critical concerns. Some centers reported reductions in elective spinal procedures, shifts in patient selection criteria, and modifications to perioperative protocols during the early pandemic [2,3]. Meanwhile, emerging evidence from general and thoracic surgery suggests that prior COVID-19 infection may influence postoperative risk. For example, in thoracic surgery, a series of patients with resolved SARS-CoV-2 infection demonstrated notable rates of postoperative respiratory complications, even when surgery was delayed several weeks after diagnosis [4]. A recent systematic review including more than 38,000 patients found that for elective surgeries conducted seven or more weeks after mild or asymptomatic COVID-19, overall 30-day mortality and serious complications returned to baseline levels compared with uninfected controls, though this depended substantially on disease severity, timing, and symptom resolution [5]. Conversely, a multicenter study in 2025 using national surgical data demonstrated that perioperative SARS-CoV-2 infection (within 30 days before or after surgery) was associated with significantly increased pulmonary complications and overall mortality [6].

In the Republic of Korea, during the study period, the government enacted rigorous nationwide infection surveillance and control measures. These policies likely minimized unrecognized or undocumented infections, reduced contamination risk, and ensured high completeness and fidelity of administrative data, making the national claims database a uniquely reliable source for epidemiologic research. Therefore, using the nationwide database of the National Health Insurance System (NHIS), we aimed to evaluate whether a history of COVID-19 is associated with an increased risk of postoperative complications, including pneumonia and spinal and implant-related infections and to identify optimal timing for elective surgery relative to a COVID-19 diagnosis.

## 2. Materials and Methods

### 2.1. Data Source

In Korea, the NHIS covers most medical expenses for all citizens, excluding cosmetic surgery, traffic accident-related services, and certain therapies lacking supporting evidence. Patients pay approximately 30% of their total medical costs directly to healthcare facilities, while the remaining 70% is reimbursed after review by the Health Insurance Review and Assessment Service. During this process, healthcare facilities must submit the appropriate diagnosis codes (based on the International Classification of Diseases, 10th Revision [ICD-10]), prescriptions, and procedural codes. All data are registered in the NHIS database and can be accessed through the National Health Insurance Sharing Service. The available dataset includes patient demographics, diagnoses, procedures, and prescription records.

This study was approved by the Institutional Review Board and Ethics Committee of our institution. The requirement for informed consent was waived owing to the retrospective nature of the study. All methods were conducted in accordance with relevant guidelines and regulations.

### 2.2. Study Population

We identified all patients diagnosed with COVID-19 between 1 January and 31 December 2020, using ICD-10 codes B342, B972, J1288, U071, U0710, U072, U089, U099, and Z29002. This group was designated the COVID-19 cohort. As a control group, we selected individuals without any COVID-19 diagnosis code during the same period, matched 3:1 by age (±1 year) and sex. Patients younger than 18 years were excluded. Baseline covariates collected included age, sex, history of diabetes mellitus and hypertension, overall comorbidity burden quantified using the Charlson Comorbidity Index (CCI), and socioeconomic status (approximated by income tertile: low, medium, or high). This matching and adjustment were intended to reduce confounding related to age, sex, comorbidity, and socioeconomic disparities—factors known to influence both COVID-19 disease severity and surgical outcomes.

### 2.3. Definition of Spinal Surgery and Exclusion Criteria

Spinal surgery was defined using specific procedure codes registered within the claims database. Eligible surgeries included discectomy (open or endoscopic), posterior or anterior instrumentation, laminectomy, corpectomy, removal of ossified ligamentum flavum, and posterior or anterior fusion across the cervical, thoracic, and lumbar levels (Table 1). To minimize confounding from emergent or non-degenerative spinal pathology, we excluded patients with diagnostic codes indicating trauma, spinal infection, or neoplasm within two weeks before or after the procedure. This approach ensured that the surgeries analyzed represented elective or planned spinal procedures and postoperative complications were less likely attributable to acute trauma, active infection, or malignancy.

### 2.4. Postoperative Complications

Postoperative complications were defined using diagnostic codes recorded after the index spinal surgery. Complications included all-cause mortality, pneumonia, respiratory failure, arrhythmia, acute cardiovascular events (angina or acute myocardial infarction), spinal and implant-related infections, deep vein thrombosis, and pulmonary thromboembolism (Table 2). Spinal and implant-related infections were identified using a predefined set of ICD-10 diagnosis codes capturing postoperative infection (T81.4), deep spinal infections such as vertebral osteomyelitis and discitis (M46), infectious spondylopathies including tuberculous and non-tuberculous etiologies (M49, A18), and infection or inflammatory reactions related to internal fixation devices or orthopedic implants (T84). Events were evaluated within predefined postoperative windows and only after the index surgery date to confirm temporal association. This claims-based method enables large-scale identification of serious complications but relies on accurate and complete coding by providers.

### 2.5. Study Design & Time Windows

**Figure 1:**
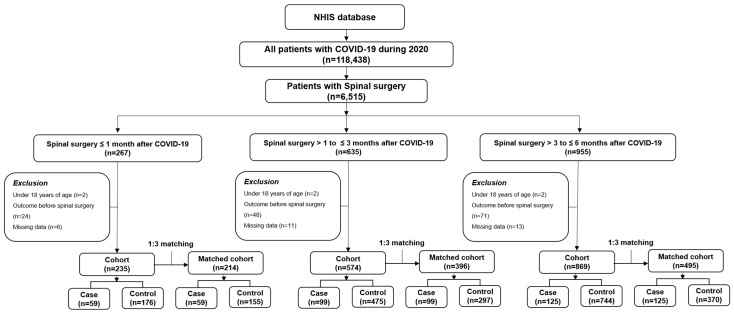
Study design.

The COVID-19 cohort was stratified based on the interval between confirmed COVID-19 diagnosis and subsequent spinal surgery. Three timing groups were defined based on the interval between COVID-19 diagnosis and the index surgical date: ≤1 month, >1–≤3 months, and >3–≤6 months. For patients with a history of COVID-19 infection, only the first eligible spinal surgery following the COVID-19 diagnosis was included in the analysis to avoid multiple observations per individual. Patients diagnosed with COVID-19 late in 2020 were followed for postoperative outcomes for up to 6 months after surgery, even when follow-up extended beyond the 2020 calendar year. The matched control group (non-COVID-19) was assigned the same index date as the corresponding case’s spinal surgery to maintain comparable calendar time distribution and account for secular trends. We compared the incidence of postoperative complications between the COVID-19 and control cohorts within each time-stratified subgroup. The study design is illustrated in Figure 1. This approach allowed us to evaluate whether proximity to COVID-19 infection was associated with differences in postoperative morbidity across timing strata.

### 2.6. Statistical Analysis

Kaplan–Meier analysis was used to assess the cumulative risk of complications in the two groups, and the log-rank test was applied to calculate *p*-values. A multivariate Cox proportional hazards model was used to analyze the hazard ratios (HRs) of complications after assessing the proportional hazards assumption using Schoenfeld residuals, with no substantial violations identified [7]. All analyses were conducted using SAS (version 9.4; SAS Institute, Cary, NC, USA) and R (version 4.0.3; R Foundation for Statistical Computing, Vienna, Austria). Statistical significance was defined as *p* < 0.05.

## 3. Results

### 3.1. Complication Comparison in Surgery ≤1 Month After COVID-19

A total of 59 patients underwent spinal surgery ≤1 month after a confirmed COVID-19 diagnosis and were compared with a matched cohort of 155 individuals without a COVID-19 diagnosis during the same period. Baseline demographics (age, sex, diabetes history, hypertension, Charlson Comorbidity Index [CCI], and income level) showed no significant differences between the two groups, indicating that observed outcome differences were associated with prior COVID-19 infection (Table 3). Notably, the HRs for developing cardiovascular disease, spinal and implant-related infections, and pneumonia within the first month postoperatively showed no significant differences between the COVID-19 and control groups.

**Figure 2:**
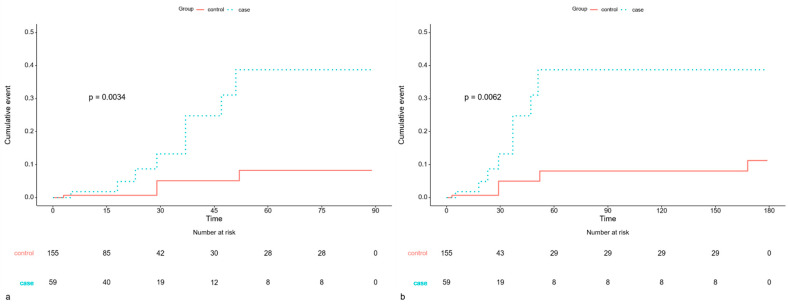
Kaplan–Meier Curve comparing the cumulative event risk of postoperative pneumonia. (**a**) Pneumonia within 3 months of surgery in patients who underwent surgery ≤1 month of COVID-19. (**b**) Pneumonia within 6 months of surgery in patients who underwent surgery ≤1 month of COVID-19.

However, in univariate analysis, the COVID-19 cohort demonstrated a markedly higher risk of developing pneumonia within 3 months post-surgery, with an HR of 5.037 (95% CI: 1.511–16.792; *p* = 0.008), indicating a fivefold increased risk in patients who underwent spinal surgery within 1 month of COVID-19 diagnosis. Multivariate analysis further confirmed that the COVID-19 group had nearly four times the hazard of developing pneumonia (HR: 3.913; 95% CI: 1.136–13.474; *p* = 0.031). The cumulative risk of pneumonia was also significantly elevated (*p* = 0.0034) (Figure 2). At 6 months postoperatively, the risk of pneumonia remained significantly higher in both univariate and multivariate analyses (HR: 4.225 and 3.121; 95% CI: 1.376–12.977 and 1.003–9.711, respectively; *p* = 0.012 and 0.049) (Table 4). In addition, the cumulative risk of developing pneumonia was higher in the COVID-19 group (*p* = 0.0062) (Figure 2).

### 3.2. Complication Comparison in Surgery >1 to ≤3 Months After COVID-19

For the cohort undergoing spinal surgery >1 to ≤3 months after COVID-19 diagnosis, 99 patients were identified and compared with 297 matched controls. Baseline demographics were similar, with the exception of a significantly higher income level in the control group (*p* = 0.021) (Table 5). No significant differences were observed between the two groups for cardiovascular complications, spinal and implant-related infections, or pneumonia within the first postoperative month.

**Figure 3:**
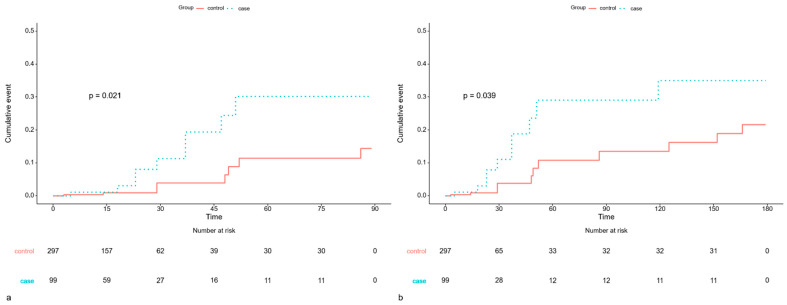
Kaplan–Meier Curve comparing the cumulative event risk of postoperative pneumonia. (**a**) Pneumonia within 3 months of surgery in patients who underwent surgery >1 to ≤3 months after COVID-19. (**b**) Pneumonia within 6 months of surgery in patients who underwent surgery >1 to ≤3 months after COVID-19.

Within 3 months after surgery, univariate analysis revealed a significantly higher risk of pneumonia in the COVID-19 group (HR: 2.915; 95% CI: 1.123–7.567; *p* = 0.028); however, this association was not statistically significant in multivariate analysis (HR: 2.548; 95% CI: 0.625–10.386; *p* = 0.192). The cumulative risk for pneumonia remained higher in the COVID-19 cohort (*p* = 0.021) (Figure 3).

**Figure 4:**
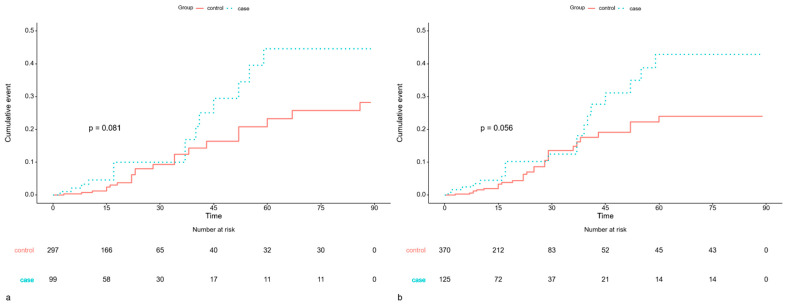
Kaplan–Meier Curve comparing the cumulative event risk of spinal and implant-related infections. (**a**) Spinal and implant-related infections within 3 months of surgery in patients who underwent surgery >1 to ≤3 months after COVID-19. (**b**) Spinal and implant-related infections within 3 months of surgery in patients who underwent surgery >3 to ≤6 months after COVID-19.

The risk of spinal and implant-related infections within 3 months was twice as high in the COVID-19 group in multivariate analysis (HR: 2.119; 95% CI: 1.034–4.344; *p* = 0.04), suggesting that prior infection may increase susceptibility to postoperative wound complications. Although the cumulative risk was higher in the COVID-19 group, this difference did not reach statistical significance (*p* = 0.081) (Figure 4).

At 6 months, univariate analysis indicated a 2.4-fold greater hazard of pneumonia in the COVID-19 group; however, the finding was not statistically significant in the multivariate model (HR: 2.315; 95% CI: 0.891–6.015; *p* = 0.085), implying that the impact of prior infection may diminish over time but remains perceptible (Table 6). The cumulative risk remained higher (*p* = 0.039) (Figure 3).

### 3.3. Complication Comparison in Surgery >3 to ≤6 Months After COVID-19

A total of 125 patients underwent spinal surgery >3 to ≤6 months of their COVID-19 diagnosis and were compared with 370 matched controls. The control group had a lower incidence of diabetes (*p* = 0.019) and higher income levels (*p* = 0.003) relative to the COVID-19 cohort (Table 7). No significant differences were observed regarding cardiovascular disease, spinal and implant-related infections, or pneumonia within the first postoperative month, suggesting that the early post-recovery period may not carry an elevated risk of these complications.

However, the risk of spinal and implant-related infections within 3 months post-surgery was twice as high in the COVID-19 cohort in multivariate analysis (HR: 2.0; 95% CI: 1.107–3.709; *p* = 0.022). Although the cumulative risk was higher in the COVID-19 group, this difference was not statistically significant (*p* = 0.056) (Figure 4).

No significant differences were found in the incidence of mortality, cardiovascular disease, arrhythmia, sepsis, or deep vein thrombosis (DVT) within 6 months postoperatively, indicating that while the initial 6 months after COVID-19 diagnosis may confer an elevated risk for specific complications such as spinal and implant-related infections, it does not appear to broadly increase other major postoperative risks (Table 8).

## 4. Discussion

This study aimed to assess the association between prior COVID-19 infection and postoperative complications in patients undergoing spinal surgery. We observed that patients with a history of COVID-19 infection were more frequently affected by pulmonary complications, particularly pneumonia, and spinal and implant-related infections after spinal surgery. These associations were most apparent within the first 3 months following infection, with similar patterns observed up to 6 months after diagnosis. Patients undergoing surgery within 1 month of COVID-19 infection demonstrated a substantially higher hazard of postoperative pneumonia compared with matched patients without prior infection. Similarly, spinal and implant-related infections were more frequently observed among patients who had COVID-19 within 3–6 months prior to surgery. These findings are consistent with the emerging literature suggesting that immune dysregulation and persistent hyperinflammation following SARS-CoV-2 infection may be associated with delayed wound healing and increased susceptibility to postoperative infections [5,6,8,9].

The observed associations are consistent with prior studies indicating that COVID-19 infection, particularly moderate to severe disease, may be associated with long-term pulmonary dysfunction. Such dysfunction significantly impairs postoperative recovery, especially in surgeries involving respiratory stress, such as spinal surgery, which often requires general anesthesia, prolonged bed rest, and, in some cases, intensive care. Several studies have demonstrated that post-COVID pulmonary complications can persist well beyond the initial infection phase [1,4,5,10]. Specifically, pneumonia, a major contributor to postoperative morbidity and mortality, has been identified as a common complication in patients recovering from COVID-19, often exacerbated by ventilator-associated pneumonia (VAP), prolonged intubation, or muscle weakness resulting from prolonged immobility [11].

Moreover, SARS-CoV-2 is known to cause vascular and endothelial damage, which can affect wound healing and promote hypercoagulation. The virus binds to angiotensin-converting enzyme 2 (ACE2) receptors, triggering a cascade of inflammatory responses [8,12]. This cascade increases oxidative stress, mitochondrial dysfunction, and thrombosis, all of which may impede proper neovascularization and wound healing and increase the risk of spinal and implant-related infections after surgery [13,14]. These mechanisms were highlighted in a recent systematic review demonstrating that COVID-19 survivors show reduced healing potential, as evidenced by delayed wound recovery and chronic inflammation [15,16]. Our findings are consistent with previous studies reporting the residual effects of post-acute COVID-19 syndrome, or “long COVID,” on both immune and vascular systems. Studies in other surgical specialties have also observed increased rates of wound infections, delayed healing, and heightened need for reoperation in patients who had COVID-19 within the preceding 6 months [17,18,19].

The timing of surgery after COVID-19 diagnosis remains a critical question in the surgical field [20]. Current guidelines suggest delaying elective surgeries for at least 7 weeks after infection to mitigate postoperative risk [21,22,23]. Our findings are broadly consistent with these recommendations but should be interpreted cautiously in the context of spinal surgery. Specifically, our analyses suggest that the timing of spinal surgery relative to COVID-19 infection may be associated with differences in postoperative complication rates, particularly for pneumonia and spinal and implant-related infections. The risk of developing these complications appears to decline gradually as the interval between diagnosis and surgery increases. However, we observed that even after 6 months, patients with prior COVID-19 continued to face increased risks compared with non-infected individuals. These observations suggest that surgery performed closer to the time of COVID-19 infection may be associated with higher postoperative complication rates, although the precision of time-stratified estimates was limited. While longer intervals between infection and surgery were generally associated with lower complication rates, residual associations were still observed in some analyses.

Given that COVID-19 affects immune function and pulmonary health, our results align with existing literature suggesting that patient-specific risk factors, such as age, pre-existing respiratory disease (e.g., COPD), and comorbidities (e.g., diabetes, hypertension), should be incorporated into preoperative risk assessments [24,25]. These factors must be integrated into surgical timing decisions, as excessive delays can result in functional decline and potentially worse overall outcomes [26].

This study offers several important clinical implications, particularly emphasizing the need for a comprehensive approach to patient management in the post-COVID era. COVID-19 has been shown to significantly affect immune function and pulmonary health, both of which are critical determinants of postoperative recovery. As observed in this study, postoperative pneumonia and spinal and implant-related infections were more frequently observed among patients with prior COVID-19 infection, even months after recovery. Given these delayed risks, surgical teams should reconsider perioperative strategies, including patient stratification and risk mitigation for high-risk groups such as older adults, patients with chronic comorbidities, and those who experienced severe COVID-19 [22,23]. These findings highlight the potential value of incorporating COVID-19 history into individualized perioperative risk assessment rather than relying on fixed timing thresholds. For instance, surgeries performed closer to the time of COVID-19 diagnosis may warrant heightened caution and individualized risk assessment, particularly for elective procedures. For patients with prolonged recovery, individualized care with enhanced postoperative monitoring may be necessary [5,6,18].

The insights from this study underscore the importance of global health preparedness in the context of surgical care. As we move into 2025, the world appears to have moved beyond the acute phases of COVID-19 through widespread vaccination and effective treatments. However, despite relaxed restrictions and lifted mandates, the potential for future pandemics or resurgences of COVID-19 or other respiratory viral infections remains a real concern. This study demonstrates that the implications of pandemics extend well beyond the immediate crisis. The long-term effects of COVID-19 on postoperative outcomes, reflected in the increased risk of pneumonia and spinal and implant-related infections, highlight the necessity of establishing frameworks addressing both immediate and lasting consequences of pandemics on surgical care.

Even as surgical volumes in spine surgery and other specialties return to pre-pandemic levels, patients who have recovered within the past 6 months may remain at increased risk for certain postoperative complications. These findings serve as a reminder that risk does not decline simply because the acute phase of a pandemic subsides. Long-term monitoring and preparedness are essential in the post-pandemic era. Future pandemics or infectious crises may disrupt medical practice at any time [27,28]. Although COVID-19 may no longer pose the same threat as at the pandemic’s peak, its endemic presence and the possibility of emerging viral threats necessitate sustained vigilance. This is particularly crucial in surgical fields, where delayed healing, infections, and complications can significantly alter outcomes. If the post-pandemic period has revealed anything, it is that healthcare systems must evolve to become more flexible and prepared for future global health crises.

This study has several limitations. First, it is a retrospective cohort study using administrative claims data, which limits the granularity of clinical details available for analysis. Detailed clinical factors, including severity of COVID-19, treatment regimens (e.g., antivirals, steroids), and intensive care requirements, were not available and could not be fully adjusted for. In particular, the inability to account for COVID-19 severity is an important limitation, as postoperative pulmonary and infectious complications are strongly influenced by the clinical severity of COVID-19 infection. Residual confounding related to unmeasured disease severity may therefore have affected the observed associations. Baseline pulmonary comorbidities (e.g., chronic obstructive pulmonary disease or asthma) and immunosuppression status could not be reliably ascertained from the claims data and were therefore not included in the analyses. Patient frailty, nutritional status, and extent of surgical trauma, which are known risk factors for postoperative complications, were also unavailable. Accordingly, a history of COVID-19 infection in this study should be interpreted as a marker of underlying vulnerability, reflecting unmeasured patient- and disease-related factors, rather than evidence of a direct causal effect on postoperative complications. Additionally, our findings are based on patients infected in early 2020, prior to widespread vaccination efforts and the emergence of later SARS-CoV-2 variants. Accordingly, the generalizability of these findings to later pandemic eras, vaccinated populations, or infections with newer variants may be limited. The dynamics of postoperative complications may differ with vaccination or newer variants, and further studies are needed to evaluate postoperative outcomes in these settings. Finally, spinal and implant-related infections were defined using ICD-10 diagnosis codes primarily capturing postoperative infection, deep spinal infections, and implant-related infections. While this approach minimizes inclusion of superficial wound infections, claims-based coding does not allow reliable differentiation of infection depth or confirmation of microbiologic severity, and some degree of outcome misclassification remains possible. Despite these limitations, this study provides valuable evidence regarding the timing of elective spinal surgery during a global pandemic and offers insights that may improve outcomes in future outbreaks.

## 5. Conclusions

In this nationwide claims-based cohort study, a history of COVID-19 infection was associated with postoperative pneumonia and spinal and implant-related infections following spinal surgery. These findings suggest that prior COVID-19 infection may be a relevant consideration in perioperative risk assessment and surgical planning. However, the observed associations should be interpreted cautiously and viewed as exploratory, highlighting the need for further studies with more detailed clinical data to better define postoperative risk following COVID-19 infection.

## Figures and Tables

**Table 1:** Procedure codes used in this study.

Surgeries for Cervical, Thoracic, Lumbar Spines	Procedure Codes
Discectomy/Endoscopic Discectomy	N1491, N1492, N1493, N1494
Posterior instrumentation	N2467, N2468, N0466, N0468, N0469, N0446, N0447, N0468, N0469
Anterior instrumentation	N0444, N0445, N0466
Corpectomy	N0451, N0452, N0453
Laminectomy	N1497, N1498, N1499, N2497, N2498, N2499
OPLL removal	N0454, N0455
Posterior fusion	N1460, N1469, N2467, N2468, N2469, N2470
Anterior fusion	N1466, N2461, N2462, N2463, N2464, N2465, N2466

OPLL, Ossification of posterior longitudinal ligament.

**Table 2:** Postoperative complications by ICD-10 codes used in this study.

Complications	Diagnostic Codes
Mortality	Mortality
Pneumonia	J12–18, J22
Respiratory failures	J95, J96, R09.2
Arrhythmias	I49
Cardiovascular diseases (AMI, Angina)	I20, I21
Sepsis	A41
Spinal and implant-related infections	M46, M49, T81.4, T84 A18
DVT	I80
PTE	I26

AMI, Acute myocardial infarction; DVT, Deep vein thrombosis; PTE, Pulmonary thromboembolism.

**Table 3:** Baseline demographics of patients with surgery ≤1 month of COVID-19 and matched cohort.

	Cases (*n* = 59)	Controls (*n* = 155)	*p*-Value
Age	64.644 (±14.598)	64.652 (±13.112)	0.997
Sex			0.759
Male	35 (59.322%)	88 (56.774%)	
Female	24 (40.678%)	67 (43.226%)	
Diabetes			0.159
No	31 (52.542%)	99 (63.871%)	
Yes	28 (47.458%)	56 (36.129%)	
Hypertension			0.759
No	24 (40.678%)	67 (43.226%)	
Yes	35 (59.322%)	88 (56.774%)	
CCI			0.194
0	15 (25.424%)	49 (31.613%)	
<3	24 (40.678%)	72 (46.452%)	
≥3	20 (33.898%)	34 (21.935%)	
Income			0.095
Low	18 (30.508%)	38 (24.516%)	
Medium	22 (37.288%)	42 (27.097%)	
High	19 (32.203%)	75 (48.387%)	

CCI, Charlson Comorbidity Index.

**Table 4:** Surgery ≤1 month after COVID-19 for developing postoperative complications by the Cox proportional hazards regression analysis.

Complications	Events (Cases/Controls)	Univariate			Multivariate		
		HR	95% CI	*p*-Value	HR	95% CI	*p*-Value
Within 1 month							
Mortality	0/0						
Cardiovascular diseases	3/9	0.892	0.241–3.295	0.863	0.383	0.083–1.768	0.219
Arrhythmias	0/0						
Spinal and implant-related infections	6/12	1.288	0.483–3.433	0.613	1.36	0.493–3.751	0.553
Respiratory failure	0/0						
Sepsis	1/0						
Pneumonia	4/3	3.454	0.773–15.434	0.105	2.735	0.54–13.858	0.224
DVT	0/0						
PTE	0/1						
Within 3 months							
Mortality	1/1	2.87	0.177–46.511	0.458			
Cardiovascular diseases	6/16	1.068	0.417–2.734	0.892	0.558	0.199–1.562	0.267
Arrhythmias	0/1						
Spinal and implant-related infections	12/14	2.102	0.97–4.554	0.06	2.196	0.972–4.965	0.059
Respiratory failure	0/0						
Sepsis	1/0						
Pneumonia	8/4	5.037	1.511–16.792	0.008 *	3.913	1.136–13.474	0.031 *
DVT	1/0						
PTE	2/1	4.818	0.436–53.208	0.199	0.201	0.018–2.262	0.194
Within 6 months							
Mortality	1/1	2.87	0.177–46.511	0.458			
Cardiovascular diseases	8/17	1.263	0.544–2.93	0.587	0.673	0.268–1.692	0.4
Arrhythmias	0/1						
Spinal and implant-related infections	12/17	1.886	0.898–3.961	0.094	1.843	0.848–4.007	0.123
Respiratory failure	0/0						
Sepsis	1/0						
Pneumonia	8/5	4.225	1.376–12.977	0.012 *	3.121	1.003–9.711	0.049 *
DVT	2/0						
PTE	2/2	2.725	0.381–19.494	0.318	1.124	0.104–12.15	0.923

Hazard rations, HRs; 95% Confidence Intervals, CIs; Deep vein thrombosis, DVT; Pulmonary thromboembolism, PTE; * indicates significance. Outcomes for which no events were observed during the study period were not included in the analyses.

**Table 5:** Baseline demographics of patients with surgery >1 to ≤3 months after COVID-19 and matched cohort.

	Cases (*n* = 99)	Controls (*n* = 297)	*p*-Value
Age	63.646 ± 15.918	64.081 ± 14.986	0.806
Sex			1
Male	57 (57.576%)	171 (57.576%)	
Female	42 (42.424%)	126 (42.424%)	
Diabetes			0.09
No	56 (56.566%)	198 (66.667%)	
Yes	43 (43.434%)	99 (33.333%)	
Hypertension			0.728
No	46 (46.465%)	132 (44.444%)	
Yes	53 (53.535%)	165 (55.556%)	
CCI			0.074
0	27 (27.273%)	112 (37.71%)	
<3	46 (46.465%)	133 (44.781%)	
≥3	26 (26.263%)	52 (17.508%)	
Income			0.021 *
Low	33 (33.333%)	67 (22.559%)	
Medium	31 (31.313%)	80 (26.936%)	
High	35 (35.354%)	150 (50.505%)	

CCI, Charlson Comorbidity Index; * indicates significance.

**Table 6:** Surgery >1 to ≤3 months after COVID-19 for developing postoperative complications by the Cox proportional hazards regression analysis.

Complications	Events (Cases/Controls)	Univariate			Multivariate		
		HR	95% CI	*p*-Value	HR	95% CI	*p*-Value
Within 1 month							
Mortality	0/0						
Cardiovascular diseases	7/8	1.646	0.831–3.26	0.153	1.505	0.501–4.515	0.466
Arrhythmias	0/0						
Spinal and implant-related infections	7/13	1.574	0.628–3.945	0.334	1.947	0.73–5.193	0.183
Respiratory failure	0/0						
Sepsis	1/0						
Pneumonia	5/4	3.394	0.911–12.65	0.069	2.548	0.625–10.386	0.192
DVT	0/0						
PTE	0/0						
Within 3 months							
Mortality	2/0						
Cardiovascular diseases	12/21	1.492	0.734–3.035	0.269	1.01	0.473–2.156	0.98
Arrhythmias	0/3						
Spinal and implant-related infections	15/22	1.78	0.922–3.437	0.086	2.119	1.034–4.344	0.04 *
Respiratory failure	0/0						
Sepsis	2/1	5.299	0.48–58.464	0.173	8.975	0.339–237.781	0.189
Pneumonia	9/8	2.915	1.123–7.567	0.028 *	2.482	0.902–6.832	0.078
DVT	0/2						
PTE	2/0						
Within 6 months							
Mortality	2/1	4.996	0.452–55.175	0.189			
Cardiovascular diseases	12/21	1.512	0.793–2.884	0.21	1.017	0.503–2.057	0.963
Arrhythmias	1/3	0.802	0.083–7.717	0.849	0.403	0.036–4.464	0.459
Spinal and implant-related infections	16/30	1.499	0.816–2.752	0.192	1.565	0.809–3.025	0.183
Respiratory failure	0/0						
Sepsis	2/2	2.712	0.382–19.256	0.319	2.551	0.272–23.887	0.412
Pneumonia	10/11	2.4	1.018–5.656	0.045 *	2.315	0.891–6.015	0.085
DVT	1/3	0.842	0.087–8.097	0.881	0.151	0.014–1.596	0.116
PTE	2/0	-	-	-	-	-	

Hazard rations, HRs; 95% Confidence Intervals, CIs; Deep vein thrombosis, DVT; Pulmonary thromboembolism, PTE; * indicates significance. Outcomes for which no events were observed during the study period were not included in the analyses.

**Table 7:** Baseline demographics of patients with surgery >3 to ≤6 months after COVID-19 and matched cohort.

	Cases (*n* = 125)	Controls (*n* = 370)	*p*-Value
Age	61.976 ± 16.336	63.4 ± 14.902	0.368
Sex			0.466
Male	69 (55.2%)	218 (58.919%)	
Female	56 (44.8%)	152 (41.081%)	
Diabetes			0.019 *
No	75 (60%)	265 (71.622%)	
Yes	50 (40%)	105 (28.378%)	
Hypertension			0.756
No	60 (48%)	185 (50%)	
Yes	65 (52%)	185 (50%)	
CCI			0.097
0	37 (29.6%)	145 (39.189%)	
<3	58 (46.4%)	159 (42.973%)	
≥3	30 (24%)	66 (17.838%)	
Income			0.003 *
Low	41 (32.8%)	82 (22.162%)	
Medium	41 (32.8%)	99 (26.757%)	
High	43 (34.4%)	189 (51.081%)	

CCI, Charlson Comorbidity Index; * indicates significance.

**Table 8:** Surgery >3 to ≤6 months after COVID-19 for developing postoperative complications by the Cox proportional hazards regression analysis.

Complications	Events (Cases/Controls)	Univariate			Multivariate		
		HR	95% CI	*p*-Value	HR	95% CI	*p*-Value
Within 1 month							
Mortality	0/0						
Cardiovascular diseases	8/13	1.718	0.712–4.146	0.229	1.362	0.552–3.362	0.502
Arrhythmias	0/1						
Spinal and implant-related infections	10/22	1.307	0.619–2.761	0.483	1.605	0.736–3.503	0.235
Respiratory failure	0/0						
Sepsis	2/0						
Pneumonia	5/8	1.732	0.566–5.302	0.336	1.532	0.486–4.831	0.466
DVT	0/0						
PTE	0/2						
Within 3 months							
Mortality	3/0						
Cardiovascular diseases	13/24	1.511	0.769–2.969	0.232	1.227	0.616–2.444	0.561
Arrhythmias	1/3	0.953	0.099–9.171	0.967	2.871	0.098–84.247	0.541
Spinal and implant-related infections	19/29	1.745	0.977–3.117	0.06	2.027	1.107–3.709	0.022 *
Respiratory failure	0/0						
Sepsis	3/0						
Pneumonia	9/12	2.014	0.847–4.785	0.113	1.957	0.796–4.81	0.143
DVT	0/1						
PTE	2/2	2.519	0.354–17.935	0.356	2.067	0.22–19.396	0.525
Within 6 months							
Mortality	3/1	8.4486	0.881–81.71	0.064			
Cardiovascular diseases	16/29	1.501	0.814–2.765	0.193	1.28	0.683–2.398	0.442
Arrhythmias	2/3	1.831	0.306–10.967	0.508	6.542	0.496–86.259	0.153
Spinal and implant-related infections	20/37	1.531	0.888–2.641	0.125	1.674	0.949–2.953	0.075
Respiratory failure	0/0						
Sepsis	3/1	8.604	0.895–82.75	0.062	7.525	0.728–77.747	0.09
Pneumonia	10/15	1.84	0.826–4.101	0.136	1.848	0.788–4.336	0.158
DVT	2/4	1.496	0.274–8.167	0.642	1.303	0.21–8.099	0.776
PTE	2/2	2.519	0.354–17.935	0.356	2.067	0.22–19.396	0.525

Hazard rations, HRs; 95% Confidence Intervals, CIs; Deep vein thrombosis, DVT; Pulmonary thromboembolism, PTE; * indicates significance. Outcomes for which no events were observed during the study period were not included in the analyses.

## Data Availability

The data that support the findings of this study are available from the Korean National Health Insurance System but restrictions apply to the availability of these data, which were used under license for the current study, and so are not publicly available. Data are, however, available from the authors upon reasonable request and with permission of the Korean National Health Insurance System.
